# The association of physical activity and carotid intima-media-thickness in adolescents—data of the prospective early vascular ageing-tyrol cohort study

**DOI:** 10.3389/fped.2025.1527132

**Published:** 2025-02-06

**Authors:** Bernhard Winder, Sophia Zollner-Kiechl, Benoît Bernar, Nina Gande, Anna Staudt, Anna K. Stock, Christoph Hochmayr, Ralf Geiger, Andrea Griesmacher, Stefan Kiechl, Ursula Kiechl-Kohlendorfer, Michael Knoflach, the Early Vascular Ageing (EVA)-Tyrol Study Group, Carmen Reiter

**Affiliations:** ^1^ Department of Vascular Surgery, Academic Teaching Hospital Feldkirch, Feldkirch, Austria; ^2^ Department of Pediatrics II, Medical University of Innsbruck, Innsbruck, Austria; ^3^ VASCage, Centre on Clinical Stroke Research, Innsbruck, Austria; ^4^ Department of Neurology, Hochzirl Hospital, Zirl, Austria; ^5^ Department of Pediatrics I, Medical University of Innsbruck, Innsbruck, Austria; ^6^ Department of Anesthesiology and Intensive Care, Medical University of Innsbruck, Innsbruck, Austria; ^7^ Department of Pediatrics III, Medical University of Innsbruck, Innsbruck, Austria; ^8^ Department of Radiology, Medical University of Innsbruck, Innsbruck, Austria; ^9^ Central Institute of Clinical Chemistry and Laboratory Medicine (ZIMCL), Medical University of Innsbruck, Innsbruck, Austria; ^10^ Department of Neurology, Medical University of Innsbruck, Innsbruck, Austria

**Keywords:** physical activity, Baecke questionnaire, carotid intima-media-thickness, cardiovascular risk factors, early vascular aging

## Abstract

**Background:**

Physical activity (PA) protects against cardiovascular disease. However, previous research has shown that high PA is associated with an increased carotid intima-media-thickness (cIMT), an independent predictor for future cardiovascular disease. Our aim was to further investigate this unexpected association with two different measurement methods of PA and two established markers for Early Vascular Ageing: cIMT and carotid-femoral pulse-wave velocity (cfPWV).

**Methods:**

The community-based Early Vascular Ageing-Tyrol cohort study included adolescents in western Austria and northern Italy. Medical examinations included anthropometric measurements, fasting blood analysis, a physician guided interview to assess lifestyle factors, measurement of cIMT and cfPWV. PA was rated by an in-person interview on the basis of average minutes of moderate- or vigorous sports per day and by the Baecke questionnaire expressed as Baecke score (BS).

**Results:**

Complete data set was available for 1,001 adolescents with a mean age of 17.8 years (standard deviation ±0.9 years). 558 (55.7%) of participants were female. cIMT was positively associated with both measures of PA in univariate (minutes sports per day: *p* < 0.001; BS: *p* < 0.001) as well as multivariable analysis adjusting for established cardiovascular risk factors (minutes sports per day: *p* = 0.001; BS: *p* = 0.002). Using cfPWV in a multivariate model an inverse correlation with the BS (*p* = 0.023) was observed, but not for minutes sports per day (*p* = 0.554).

**Conclusion:**

In our large community-based cohort of adolescents, PA was associated with an increased cIMT but shows a trend towards lower aortic stiffness measured by cfPWV. We hypothesize that the association of PA with increased cIMT is not caused by early atherosclerotic vessel wall changes but is rather a physiologic adaptive process of the vessel wall.

**Trial Registration Number:**

The EVA-Tyrol Study has been registered at clinicaltrials.gov under NCT03929692 since April 29, 2019.

## Introduction

Cardio- and cerebrovascular disease (CVD) remains the leading cause of death in Europe with atherosclerosis as the main underlying condition (1, 2). Observations from autopsy studies demonstrate a significant association between cardiovascular risk factors (cvRF) and atherosclerotic lesions even in early life (3). Based on this finding, it is well established that cvRF are predictive of future development of CVD (4). Great importance must therefore be given to the prevention of cvRF from a young age on.

In several studies, physical activity (PA) has been demonstrated to be one of the essential preventive interventions for atherosclerosis (5, 6). To investigate the effect of PA on atherosclerotic vascular changes, various studies have been conducted in adult study cohorts (7, 8). Regarding children and adolescents, however, available evidence is still scarce. Carotid intima media thickness (cIMT) and carotid-femoral pulse-wave velocity (cfPWV) offer an opportunity to measure early atherosclerosis—or more appropriately termed early vascular ageing (EVA)—non-invasively *in vivo* (9–15). These markers have been associated with various cvRF at young age (16, 17).

Contrary to what might be expected, we and others described a positive association between PA and cIMT (17–21) which could be interpreted that high PA could have unfavorable effects on vascular ageing in children and adolescents. To further investigate this primary unintuitive outcome, the aim of this work is to perform a depth secondary analysis of the large and homogenous EVA-Tirol study population of two assessments of PA [self-reported minutes of sports per day and the validated Baecke Score (BS) (22)] with two independent measures of EVA (cIMT and cfPWV) with additional parameters of PA and EVA.

## Materials and methods

Data that support the findings of this study are available from the corresponding authors upon reasonable request after signing the appropriate data transfer agreement.

### Study design and participants

The EVA-Tyrol cohort study is a prospective cohort study aiming to assess CVD risk profiles in adolescents throughout North, East (Austria) and South Tyrol (Italy) and testing a pragmatic health promotion program. The study was conducted between May 2015 and July 2018 at local schools and companies (vocational schools). Students in 9th or 10th grade (target age 14–16 years) and apprentices of the same age were invited to participate at baseline examinations, which were conducted between May 2015 and December 2016 and included systematic health screening with a particular focus on EVA. Subsequently, a nonrandomized controlled lifestyle intervention was implemented to improve cardiovascular health. Approximately two years later (target age 16–18 years), a follow-up survey was performed between August 2017 and July 2018. Simultaneously, another group of adolescents (target age 16–18 years) took part as participants in the control group. To achieve a representative sample of adolescents homogenous in age, participants of the intervention group as well as the control group were included in the present analysis.

A detailed description of the EVA-Tyrol cohort study protocol has been published (23). The study was approved by the local ethics committee of the Medical University of Innsbruck (approval number AN 2015-0005 345/4.13) and was conducted in agreement with the Declaration of Helsinki. The trial has been registered at clinicaltrials.gov (NCT03929692). All participants provided a written informed consent or if the participants had not attained age of majority, the consent was additionally provided by a parent or legal guardian. Data is available upon reasonable request at the corresponding author after signing an appropriate data transfer agreement and providing a specific ethics vote for secondary analysis.

### Anthropometry

For measurement of height and weight participants wore light indoor clothes and no shoes. Height was determined with a Harpenden stadiometer (Holtain, Crymych, United Kingdom), and weight with a calibrated medical precision scale. Body mass index (BMI) was calculated as ratio of the body weight in kilograms divided by the square of height in meters. Systolic and diastolic blood pressures were calculated as the mean of three independent measurements on both upper arms in a sitting position after a 5-min seated rest (automated oscillometric device OMRON M4-I, Omron Healthcare Co., Lake Forest, Illinois, USA).

### Lifestyle risk factors

We assessed behavioral risk factors by a face-to-face physician-guided medical interview with questionnaires adapted from the Atherosclerosis Risk Factors in Female Youngsters (ARFY), Atherosclerosis Risk Factors in Male Youngsters (AMRY) and Bruneck studies (24–26). PA was obtained as minutes per day of moderate- or vigorous sports (i.e., leading to an increased heart rate and/or sweating) in a face-to-face interview and using the Baecke questionnaire. The Baecke questionnaire assesses current PA according to three different domains: work (school), leisure time, and practice of sports. Each domain consists of several questions rated on a four to five-point Likert scale, ranging from never to always or very often. For the two most frequently reported sport activities, additional questions query the type of activity, number of months per year and hours per week of participation. Summing of the separately calculated domain scores results in the continuous overall and unitless Baecke Score (BS) with higher values representing a higher physical activity (27). Cigarette pack years (PY) were calculated by multiplying the number of packs of cigarettes smoked per day by the number of years the person has smoked.

### Laboratory methods

Blood samples were drawn after an overnight fasting period of more than eight hours, immediately cooled, delivered, and analyzed within 24 h after venipuncture by the Central Institute for Medical and Chemical Laboratory Diagnosis of the Medical University of Innsbruck, Austria. Total cholesterol, low-density lipoprotein cholesterol and high-density lipoprotein cholesterol (HDL-C) were assessed by a standard enzymatic colorimetric assay (Cobas 8000, Roche Diagnostics, Rotkreuz, Switzerland). Serum glucose was analyzed by a hexokinase method (Cobas 8000, Roche Diagnostics, Rotkreuz, Switzerland). Measurement of Alanine aminotransferase (ALT) was performed according to the recommendations of the international federation of Clinical Chemistry and Laboratory Medicine (Cobas 8000, Roche Diagnostics, Rotkreuz, Switzerland).

### High-resolution ultrasound

cIMT was assessed in the supine position using a high-resolution linear ultrasound probe (6.0–13.0 MHz, GE 12l-RS) on a Vivid q ultrasound device, (both General Electric Healthcare, Chicago, Illinois, USA). Three representative measurements were taken on both sides in longitudinal images on the far-wall of the distal 4 cm of the common carotid arteries. A single rater (A.S.), experienced in ultrasound techniques and blinded to the clinical characteristics of the study participants, performed all measurements on digitally stored images being unaware of the clinical characteristics. The mean from all six measurements was used as cIMT for the present analysis. For 103 randomly selected participants, repeated measurements were performed (by M.K.) to obtain an inter-observer reproducibility, which yielded a Pearson correlation coefficient of 0.634, indicating moderate agreement that is consistent with the measurement precision for cIMT of ultrasound.

### Carotid-femoral pulse-wave velocity

To measure cfPWV participants were examined in the supine resting position using the automated Complior Analyze® (Alam Medical, Vincennes, France) according to the manufactureŕs instructions and in accordance with previously validated protocols (28, 29). Carotid and femoral artery pulse waves were recorded simultaneously by applanation tonometry, with two transducers placed transcutaneously on the ipsilateral carotid and femoral artery. The cfPWV was then automatically calculated as the travel distance divided by the transit time of the carotid-femoral pulse waves [travel distance (meters)/transit time (seconds)]. Pulse wave transit time was determined from ten consecutive pulse waves of artefact-free cardiac cycles, measuring the mean of time delay between the base of the two waveforms in the carotid and the femoral arteries. Travel distance was measured within 0.1 cm over the body surface with a non-stretchable tape between the two recording sites. The mean of two representative measurements was used as cfPWV.

### Statistical analysis

Statistical analysis was performed using SPSS version 29.0 (IBM Corporation, Armonk, New York, USA). Our primary outcome parameters were cIMT and cfPWV. Participants without valid cIMT and/or cfPWV measurements were excluded from our analysis. Study cohort characteristics are presented as number (percentage), mean ± standard deviation or median (*Q*_25_–*Q*_75_). The comparative analyses males vs. females were performed using the Welch-t-test or the Chi-Square test as applicable. Univariate analyses were assessed with Spearman product-moment correlation coefficient. For multivariable linear regression analysis, variables known to affect cIMT in our study cohort (age, sex, systolic blood pressure, cigarette pack years, ALT) (17) and other common cvRF (BMI, HDL-C and fasting serum glucose) were included in the model. PA was integrated using minutes sports per day and the BS. Variables not meeting the assumptions of a normal distribution were log_e_-transformed (BMI, systolic blood pressure, PY, ALT, HDL-C). All models were tested for collinearity by variance inflation factors. The conditions of linearity of the relationship between dependent and independent variables as well as homoscedasticity, independence and normality of the errors were tested and fulfilled. Multiple linear regression models were finally calculated separately to assess the impact of the PA on cIMT and cfPWV. *p*-Values <0.05 were considered statistically significant.

## Results

**Figure 1:**
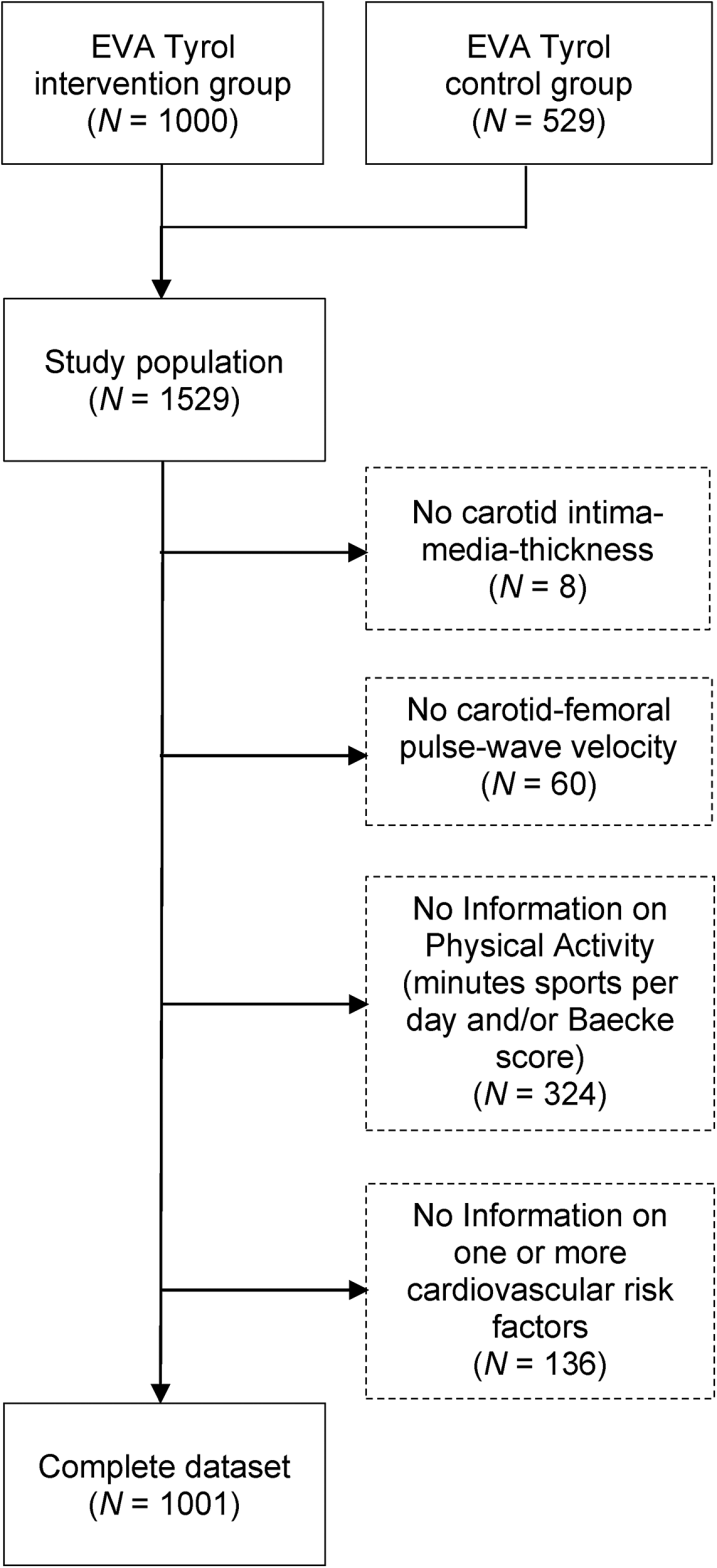
EVA-Tyrol study participation flow chart. From: The association of physical activity and carotid intima-media-thickness in adolescents—Data of the prospective Early Vascular Ageing (EVA)-Tyrol cohort study.

The analysis was performed with 1,000 participants from the intervention group (all participants who completed the follow-up examination) and 529 participants from the control group. After excluding participants with an incomplete data set, 1,001 (65.5%) individuals could be included in the analyses below (see flow chart in Figure 1). There were no significant differences between participants in- and excluded in the present evaluation (data not shown). 558 (55.7%) of the participants were female. The mean age of the study population at the time of the examinations was 17.8 years (standard deviation 0.9, range 16–23 years). The mean cIMT was 417.8 (± 49.0) µm [female: 407.6 (±44.5) µm, male: 430.7 (±51.5) µm; *p* < 0.001]. The mean value of cfPWV was 6.1 (±0.8) m/s [females: 6.0 (±0.7) m/s, males: 6.3 (±0.9) m/s; *p* < 0.001].

The median daily PA was 40.0 (25.0–60.0) min, with a significant difference (*p* < 0.001) between female: 30.0 (20.0–60.0) min and male: 51.4 (30.0–70.0) min study participants. Taking the Baecke score as the second parameter for PA [median of the entire study population: 7.9 (7.1–8.6)], this gender-specific difference [females: 7.8 (7.0–8.5), males: 8.0 (7.3–9.0)] was confirmed (*p* < 0.001). Further details of the participants can be found in Table 1.

**Table 1:** Characteristics of study population.

Study population	Total	Male	Female	*p-*value
Participants	1,001	443 (44.3%)	558 (55.7%)	
Demographics				
Age (years)	17.8 (±0.9)	17.9 (±0.9)	17.7 (±0.9)	0.006
Anthropometrics				
Height (cm)	172.5 (166.0–179.5)	180.0 (176.0–185.0)	167.0 (63.0–171.0)	<0.001
Weight (kg)	64.8 (58.4–73.8)	71.0 (64.4–78.6)	60.6 (55.0–67.3)	<0.001
BMI (kg/m²)	21.7 (19.9–24.0)	21.8 (20.0–24.1)	21.6 (19.8–23.9)	0.530
Physical activity				
Sports per day (min)	40.0 (25.0–60.0)	51.4 (30.0–70.0)	30.0 (20.0–60.0)	<0.001
Baecke score	7.9 (7.1–8.6)	8.0 (7.3–9.0)	7.8 (7.0–8.5)	<0.001
Blood pressure				
Systolic blood pressure (mmHg)	121.3 (114.0–130.7)	127.7 (120.0–137.0)	117.7 (100.0–123.7)	<0.001
Diastolic blood pressure (mmHg)	70.3 (65.0–76.0)	70.0 (64.7–76.0)	70.7 (65.7–76.0)	0.246
Smoking				
Current Smoker	230 (23.0%)	95 (21.4%)	135 (24.2%)	0.314
Ever Smoker	332 (33.2%)	140 (31.6%)	192 (34.4%)	0.349
Pack-years	0.000 (0.000–0.004)	0.000 (0.000–0.002)	0.000 (0.000–0.007)	0.240
Lipids				
Total cholesterol (mg/dl)	159.0 (139.0–179.5)	150.0 (132.0–169.0)	166.0 (148.0–187.0)	<0.001
HDL-C (mg/dl)	56.0 (49.0–66.0)	52.0 (46.0–59.0)	60.0 (53.0–71.0)	<0.001
LDL-C (mg/dl)	93.0 (78.0–111.0)	90.0 (75.0–107.0)	94.5 (81.0–114.0)	<0.001
Metabolic markers				
Alanine transaminase (U/L)	17.0 (13.0–22.0)	19.0 (15.0–26.0)	15.0 (12.0–19.0)	<0.001
Fasting serum glucose (mg/dl)	78.0 (71.0–84.0)	80.0 (74.0–87.0)	75.0 (69.0–81.0)	<0.001
Early vascular ageing				
cIMT (µm)	417.8 (±49.0)	430.7 (±51.5)	407.6 (±44.5)	<0.001
cfPWV (m/sec)	6.1 (±0.8)	6.3 (±0.9)	6.0 (±0.7)	<0.001

From: The association of physical activity and carotid intima-media-thickness in adolescents—Data of the prospective Early Vascular Ageing (EVA)-Tyrol cohort study.

Values are given as number (%), mean (±standard deviation) or median (*Q*_25_–*Q*_75_).

*p*-Value comparison males vs. females using the Welch-t-test or the Chi-Square test as applicable.

BMI, body mass index; HDL-C, high-density lipoprotein cholesterol; LDL-C, low-density lipoprotein cholesterol; cIMT carotid intima-media-thickness; cfPWV, carotid-femoral pulse-wave velocity.

cIMT was positively correlated with PA measured as minutes sports per day (*r* = 0.152, *p* < 0.001) or by the BS (*r* = 0.130, *p* < 0.001) indicating that a higher PA was associated with an increased vessel wall thickness. This association remained robust in the multivariable regression analysis adjusting for sex, age, BMI, systolic blood pressure, ALT, HDL-C, PY and fasting serum glucose (Table 2).

**Table 2:** Univariate and multivariate analyses results.

	Univariable correlation coefficient (*p-*value)	Multivariable unstandardized regression coefficient, 95% confidence interval (*p-*value)
Carotid intima-media-thickness (µm)		
Physical Activity (minutes sports per day)	*r* = 0.152 (< 0.001)	*B* = 0.140, 95% CI: 0.054–0.227 (0.001)
Physical Activity (Baecke score)	*r* = 0.130 (< 0.001)	*B* = 3.869, 95% CI: 1.455–6.283 (0.002)
Carotid-femoral pulse-wave velocity (m/sec)		
Physical Activity (minutes sports per day)	*r* = 0.065 (0.040)	*B* = 0.000, 95% CI: −0.001 to 0.002 (0.554)
Physical Activity (Baecke score)	*r* = −0.054 (0.090)	*B* = −0.045, 95% CI: −0.084 to −0.006 (0.023)

From: The association of physical activity and carotid intima-media-thickness in adolescents—Data of the prospective Early Vascular Ageing (EVA)-Tyrol cohort study.

The univariable analyses were performed using the Spearman product moment.

Multivariable linear regression model included age, sex, body mass index, systolic blood pressure, cigarette pack years, alanine transaminase, high-density lipoprotein cholesterol and fasting serum glucose.

cfPWV was positively correlated with PA measured in minutes sports per day (*r* = 0.065, *p* = 0.040). This association was rendered insignificant after adjustment for sex, age and cvRF (*p* = 0.554). When using the BS as measurement for PA no correlation (univariable) was found with cfPWV, yet in a multivariable linear regression analysis adjusting for the factors above, a weak inverse association was found with an unstandardized regression coefficient (95% confidence interval) of −0.045 (−0.084 to −0.006) (*p* = 0.023) (Table 2).

## Discussion

We have previously reported a positive association between PA measured in minutes sports per day and cIMT in this cohort (17). The novelty of our present analysis is to reproduce this finding with the BS as an alternative parameter for PA providing a more complete representation of activity behavior and explore the effect of PA on another parameter of EVA.

It is counter-intuitive that a higher PA is associated with a higher cIMT (i.e., vessel wall thickness), an established surrogate marker for atherosclerosis and future risk of CVD (30, 31), especially as PA has been proven to have a protective effect against the development of CVD (32).

Previous studies have seen similar effects (18–21), but other studies also have found an inverse association of PA on cIMT (33–35). Yet the latter studies have examined considerably younger children (33) or have demonstrated a modest reduction in cIMT after a PA intervention program in obese adolescents and not a cross-sectional association (34, 35).

Current data on the pathophysiology of the relationship between PA and cIMT are very limited. One possible explanation is that increased blood flow during PA leads to an elevated shear stress on the arterial vessel wall, and thereby to an release of prostacyclin and an enhanced synthesis of nitric oxide by the endothelial nitric oxide synthase (36–38). The resulting dilation of the carotid artery leads to a stimulation of endothelial progenitor cells with subsequent arteriogenesis and endothelial repair mechanisms (39, 40).

An alternative explanation would be that moderate/vigorous PA leads to adaptive processes in the Tunica media and the vascular smooth muscle cells, collagen and elastin localized there. Blood pressure peaks during PA result in a mechanical stimulus that leads to a change in the proportion of smooth muscle cells in the intimal layer and to a relative increase in the ratio of collagen to elastin in the media layer to maintain “tensional homeostasis” (41). An over-all increase in cIMT would therefore not be due to an atherosclerotic change in the vessel wall, but rather a physiological adaptive process (21).

When analyzing another parameter for EVA—the cfPWV, reflecting primarily the stiffness of the aorta (with higher values indicating an accelerated EVA) (15, 42)—we have found no independent association between PA measured in minutes per day and a small inverse (i.e., favourable), independent effect of PA assessed by the BS. Therefore, we cannot confirm that PA leads to an increased EVA in the aorta but might already have some protective properties. Other measures of arterial elasticity or stiffness used in published cohorts were not or positively influenced by PA (19, 43–45).

As autopsy studies have demonstrated that earliest morphological changes of arteriosclerosis occur in the vessel wall of the abdominal aorta, even before those in the carotid artery (46). This observation is supported by the study results of Weberruß et al, Pahkala et al. and Königsstein et al, who found in their analyses of adolescents the first signs of EVA earlier in the abdominal aorta (measured as aortic IMT) than in the carotid arteries (measured as cIMT) (43, 47, 48). It is counter-intuitive that PA leads to a premature atherosclerotic vessel wall thickening in the common carotid arteries and to the opposite effect in the aorta (reduced aortic stiffness). Considering these findings, our results support the hypothesis of Baumgartner et al. that the increased IMT in the carotid arteries of physical active adolescents are not due to atherosclerotic wall changes but rather reflect physiologic adaptive processes in the vessel wall (20).

To the best of our knowledge this is the first study to specifically address the issue of a positive association between PA and cIMT in a community-based cohort of adolescents. The strengths of our studies include the large and homogeneous study cohort of young participants and the high quality of the assessment of cvRF and health-specific lifestyle parameters through structured, physician-guided examinations and interviews. The size of our study cohort and the structured assessment of cvRF assessed, enabled a comprehensive consideration of relevant confounders.

There are also some limitations. Even though we have used well-established assessment instruments for PA we cannot exclude a recall bias, and we do not have objective measurements e.g., by accelerometers, pedometers or heart rate monitors. In addition, we had to exclude approximately one third of our population due to missing variables (see flow chart in Figure 1). We have also recruited a sole Middle-European cohort, and our findings may not be generalizable to other communities and populations. Finally, we recognize that the interpretation of our data is—in part—speculative. However, like others before us (19, 21), we must point out that the described topic of the effect of PA on EVA is still under-researched, especially in physiological terms. Valuable insights could be gained from large prospective cohorts monitoring the development of EVA as well as from basic research focusing on vascular remodeling under high PA in healthy blood vessels.

## Conclusion

PA is associated with an increased cIMT but showed a trend to a reduced aortic stiffness as measured by cfPWV. Therefore, based on our results and the currently available evidence, we conclude that it is unlikely that a high PA leads to an accelerated EVA. It is very tempting to speculate that PA leads to a physiologic adaptive processes in the cIMT that do not resemble early atherosclerosis, yet in the long run, protects from vascular ageing, atherosclerosis and cardiovascular events. Further studies are needed.

## Data Availability

Data is available upon reasonable request at the corresponding author after signing an appropriate data transfer agreement and providing a specific ethics vote for secondary analysis.

## Group members of the Early Vascular Ageing (EVA)-Tyrol Study Group

Carmen Reiter, Christina Schreiner Julia Klingenschmid, Julia Marxer, Martina Kothmayer and Maximilian Pircher, Department of Pediatrics II, Medical University of Innsbruck, Anichstrasse 35, A-6020 Innsbruck, Austria; Manuela Bock-Bartl, VASCage, Centre on Clinical Stroke Research, Adamgasse 23, A-6020 Innsbruck, Austria; Mandy Asare, Maximilian Bohl and Raimund Pechlaner, Department of Neurology, Medical University of Innsbruck, Anichstrasse 35, A-6020 Innsbruck, Austria.

## References

[1] Townsend N Kazakiewicz D Lucy Wright F Timmis A Huculeci R Torbica A Epidemiology of cardiovascular disease in Europe. Nat Rev Cardiol. (2022) 19(2):133–43. 10.1038/s41569-021-00607-3 34497402

[2] Libby P Ridker PM Hansson GK. Progress and challenges in translating the biology of atherosclerosis. Nature. (2011) 473(7347):317–25. 10.1038/nature10146 21593864

[3] Berenson GS Srinivasan SR Bao W Newman WP III Tracy RE Wattigney WA. Association between multiple cardiovascular risk factors and atherosclerosis in children and young adults. The Bogalusa Heart Study. N Engl J Med. (1998) 338(23):1650–6. 10.1056/NEJM199806043382302 9614255

[4] Berenson GS. Childhood risk factors predict adult risk associated with subclinical cardiovascular disease. The Bogalusa Heart Study. Am J Cardiol. (2002) 90(10c):3l–7l. 10.1016/s0002-9149(02)02953-3 12459418

[5] Yang J Cao RY Gao R Mi Q Dai Q Zhu F. Physical exercise is a potential “Medicine” for atherosclerosis. Adv Exp Med Biol. (2017) 999:269–86. 10.1007/978-981-10-4307-9_15 29022268

[6] Cooper DM Radom-Aizik S. Exercise-associated prevention of adult cardiovascular disease in children and adolescents: monocytes, molecular mechanisms, and a call for discovery. Pediatr Res. (2020) 87(2):309–18. 10.1038/s41390-019-0581-7 31649340 PMC11177628

[7] Tanaka H Dinenno FA Monahan KD Clevenger CM DeSouza CA Seals DR. Aging, habitual exercise, and dynamic arterial compliance. Circulation. (2000) 102(11):1270–5. 10.1161/01.cir.102.11.1270 10982542

[8] Gando Y Yamamoto K Kawano H Murakami H Ohmori Y Kawakami R Attenuated age-related carotid arterial remodeling in adults with a high level of cardiorespiratory fitness. J Atheroscler Thromb. (2011) 18(3):248–54. 10.5551/jat.6924 21123955

[9] Willeit P Tschiderer L Allara E Reuber K Seekircher L Gao L Carotid intima-media thickness progression as surrogate marker for cardiovascular risk: meta-analysis of 119 clinical trials involving 100667 patients. Circulation. (2020) 142(7):621–42. 10.1161/CIRCULATIONAHA.120.046361 32546049 PMC7115957

[10] O'Leary DH Bots ML. Imaging of atherosclerosis: carotid intima-media thickness. Eur Heart J. (2010) 31(14):1682–9. 10.1093/eurheartj/ehq185 20542989

[11] Raitakari OT Juonala M Kähönen M Taittonen L Laitinen T Mäki-Torkko N Cardiovascular risk factors in childhood and carotid artery intima-media thickness in adulthood: the cardiovascular risk in young Finns study. Jama. (2003) 290(17):2277–83. 10.1001/jama.290.17.2277 14600186

[12] Laurent S. Defining vascular aging and cardiovascular risk. J Hypertens. (2012) 30(Suppl):S3–8. 10.1097/HJH.0b013e328353e501 23124102

[13] Nilsson PM Lurbe E Laurent S. The early life origins of vascular ageing and cardiovascular risk: the EVA syndrome. J Hypertens. (2008) 26(6):1049–57. 10.1097/HJH.0b013e3282f82c3e 18475139

[14] Magnussen CG Smith KJ Juonala M. When to prevent cardiovascular disease? As early as possible: lessons from prospective cohorts beginning in childhood. Curr Opin Cardiol. (2013) 28(5):561–8. 10.1097/HCO.0b013e32836428f4 23928921

[15] Vlachopoulos C Aznaouridis K Stefanadis C. Prediction of cardiovascular events and all-cause mortality with arterial stiffness: a systematic review and meta-analysis. J Am Coll Cardiol. (2010) 55(13):1318–27. 10.1016/j.jacc.2009.10.061 20338492

[16] Kiechl SJ Staudt A Stock K Gande N Bernar B Hochmayr C Predictors of carotid intima-Media thickness progression in adolescents-the EVA-tyrol study. J Am Heart Assoc. (2021) 10(18):e020233. 10.1161/JAHA.120.020233 34482715 PMC8649517

[17] Staudt A Stock K Gande N Bernar B Hochmayr C Pechlaner R Impact of lifestyle and cardiovascular risk factors on early atherosclerosis in a large cohort of healthy adolescents: the early vascular ageing (EVA)-tyrol study. Atherosclerosis. (2020) 305:26–33. 10.1016/j.atherosclerosis.2020.05.011 32603950

[18] Ascenso A Palmeira A Pedro LM Martins S Fonseca H. Physical activity and cardiorespiratory fitness, but not sedentary behavior, are associated with carotid intima-media thickness in obese adolescents. Eur J Pediatr. (2016) 175(3):391–8. 10.1007/s00431-015-2654-x 26490566

[19] Baumgartner L Weberruß H Engl T Schulz T Oberhoffer-Fritz R. Exercise training duration and intensity are associated with thicker carotid intima-media thickness but improved arterial elasticity in active children and adolescents. Front Cardiovasc Med. (2021) 8:618294. 10.3389/fcvm.2021.618294 34307488 PMC8295565

[20] Baumgartner L Weberruß H Appel K Engl T Goeder D Oberhoffer-Fritz R Improved carotid elasticity but altered central hemodynamics and carotid structure in young athletes. Front Sports Act Living. (2021) 3:633873. 10.3389/fspor.2021.633873 33791599 PMC8005716

[21] Ried-Larsen M Grøntved A Kristensen PL Froberg K Andersen LB. Moderate-and-vigorous physical activity from adolescence to adulthood and subclinical atherosclerosis in adulthood: prospective observations from the European youth heart study. Br J Sports Med. (2015) 49(2):107–12. 10.1136/bjsports-2013-092409 23584827

[22] Tebar WR Ritti-Dias RM Fernandes RA Damato TMM Barros MVG Mota J Validity and reliability of the baecke questionnaire against accelerometer-measured physical activity in community dwelling adults according to educational level. PLoS One. (2022) 17(8):e0270265. 10.1371/journal.pone.0270265 35969609 PMC9377570

[23] Bernar B Gande N Stock KA Staudt A Pechlaner R Geiger R The tyrolean early vascular ageing-study (EVA-tyrol): study protocol for a non-randomized controlled trial: effect of a cardiovascular health promotion program in youth, a prospective cohort study. BMC Cardiovasc Disord. (2020) 20(1):59. 10.1186/s12872-020-01357-9 32024473 PMC7001281

[24] Knoflach M Kiechl S Kind M Said M Sief R Gisinger M Cardiovascular risk factors and atherosclerosis in young males: ARMY study (atherosclerosis risk-factors in male youngsters). Circulation. (2003) 108(9):1064–9. 10.1161/01.CIR.0000085996.95532.FF 12952827

[25] Knoflach M Kiechl S Penz D Zangerle A Schmidauer C Rossmann A Cardiovascular risk factors and atherosclerosis in young women: atherosclerosis risk factors in female youngsters (ARFY study). Stroke. (2009) 40(4):1063–9. 10.1161/STROKEAHA.108.525675 19211497

[26] Kiechl S Willeit J. The natural course of atherosclerosis. Part I: incidence and progression. Arterioscler Thromb Vasc Biol. (1999) 19(6):1484–90. 10.1161/01.atv.19.6.1484 10364079

[27] Baecke JA Burema J Frijters JE. A short questionnaire for the measurement of habitual physical activity in epidemiological studies. Am J Clin Nutr. (1982) 36(5):936–42. 10.1093/ajcn/36.5.936 7137077

[28] Stea F Bozec E Millasseau S Khettab H Boutouyrie P Laurent S. Comparison of the complior analyse device with sphygmocor and complior SP for pulse wave velocity and central pressure assessment. J Hypertens. (2014) 32(4):873–80. 10.1097/HJH.0000000000000091 24509122

[29] Rajzer MW Wojciechowska W Klocek M Palka I Brzozowska-Kiszka M Kawecka-Jaszcz K. Comparison of aortic pulse wave velocity measured by three techniques: complior, SphygmoCor and arteriograph. J Hypertens. (2008) 26(10):2001–7. 10.1097/HJH.0b013e32830a4a25 18806624

[30] de Groot E Hovingh GK Wiegman A Duriez P Smit AJ Fruchart JC Measurement of arterial wall thickness as a surrogate marker for atherosclerosis. Circulation. (2004) 109(23 Suppl 1):Iii33–8. 10.1161/01.CIR.0000131516.65699.ba 15198964

[31] Lamotte C Iliescu C Libersa C Gottrand F. Increased intima-media thickness of the carotid artery in childhood: a systematic review of observational studies. Eur J Pediatr. (2011) 170(6):719–29. 10.1007/s00431-010-1328-y 20978785

[32] Visseren FLJ Mach F Smulders YM Carballo D Koskinas KC Bäck M 2021 ESC guidelines on cardiovascular disease prevention in clinical practice. Eur Heart J. (2021) 42(34):3227–337. 10.1093/eurheartj/ehac458 34458905

[33] Idris NS Evelein AM Geerts CC Sastroasmoro S Grobbee DE Uiterwaal CS. Effect of physical activity on vascular characteristics in young children. Eur J Prev Cardiol. (2015) 22(5):656–64. 10.1177/2047487314524869 24526797

[34] Meyer AA Kundt G Lenschow U Schuff-Werner P Kienast W. Improvement of early vascular changes and cardiovascular risk factors in obese children after a six-month exercise program. J Am Coll Cardiol. (2006) 48(9):1865–70. 10.1016/j.jacc.2006.07.035 17084264

[35] García-Hermoso A González-Ruiz K Triana-Reina HR Olloquequi J Ramírez-Vélez R. Effects of exercise on carotid arterial wall thickness in obese pediatric populations: a meta-analysis of randomized controlled trials. Child Obes. (2017) 13(2):138–45. 10.1089/chi.2016.0265 28061145

[36] Tinken TM Thijssen DH Hopkins N Dawson EA Cable NT Green DJ. Shear stress mediates endothelial adaptations to exercise training in humans. Hypertension. (2010) 55(2):312–8. 10.1161/HYPERTENSIONAHA.109.146282 20048193

[37] Padilla J Simmons GH Bender SB Arce-Esquivel AA Whyte JJ Laughlin MH. Vascular effects of exercise: endothelial adaptations beyond active muscle beds. Physiology (Bethesda). (2011) 26(3):132–45. 10.1152/physiol.00052.2010 21670160 PMC3286126

[38] Green DJ Hopman MT Padilla J Laughlin MH Thijssen DH. Vascular adaptation to exercise in humans: role of hemodynamic stimuli. Physiol Rev. (2017) 97(2):495–528. 10.1152/physrev.00014.2016 28151424 PMC5539408

[39] Whyte JJ Laughlin MH. The effects of acute and chronic exercise on the vasculature. Acta Physiol (Oxf). (2010) 199(4):441–50. 10.1111/j.1748-1716.2010.02127.x 20353494 PMC3059589

[40] Schmidt W Endres M Dimeo F Jungehulsing GJ. Train the vessel, gain the brain: physical activity and vessel function and the impact on stroke prevention and outcome in cerebrovascular disease. Cerebrovasc Dis. (2013) 35(4):303–12. 10.1159/000347061 23594423

[41] Bertovic DA Waddell TK Gatzka CD Cameron JD Dart AM Kingwell BA. Muscular strength training is associated with low arterial compliance and high pulse pressure. Hypertension. (1999) 33(6):1385–91. 10.1161/01.hyp.33.6.1385 10373221

[42] Urbina EM Williams RV Alpert BS Collins RT Daniels SR Hayman L Noninvasive assessment of subclinical atherosclerosis in children and adolescents: recommendations for standard assessment for clinical research: a scientific statement from the American Heart Association. Hypertension. (2009) 54(5):919–50. 10.1161/HYPERTENSIONAHA.109.192639 19729599

[43] Pahkala K Laitinen TT Heinonen OJ Viikari JS Rönnemaa T Niinikoski H Association of fitness with vascular intima-media thickness and elasticity in adolescence. Pediatrics. (2013) 132(1):e77–84. 10.1542/peds.2013-0041 23753102

[44] Ried-Larsen M Grøntved A Froberg K Ekelund U Andersen LB. Physical activity intensity and subclinical atherosclerosis in danish adolescents: the European youth heart study. Scand J Med Sci Sports. (2013) 23(3):e168–77. 10.1111/sms.12046 23336399

[45] Weberruß H Pirzer R Schulz T Böhm B Dalla Pozza R Netz H Reduced arterial stiffness in very fit boys and girls. Cardiol Young. (2017) 27(1):117–24. 10.1017/S1047951116000226 27020795

[46] McGill HC J McMahan CA Herderick EE Tracy RE Malcom GT Zieske AW Effects of coronary heart disease risk factors on atherosclerosis of selected regions of the aorta and right coronary artery. PDAY research group. Pathobiological determinants of atherosclerosis in youth. Arterioscler Thromb Vasc Biol. (2000) 20(3):836–45. 10.1161/01.atv.20.3.836 10712411

[47] Weberruß H Pirzer R Böhm B Elmenhorst J Pozza RD Netz H Increased intima-media thickness is not associated with stiffer arteries in children. Atherosclerosis. (2015) 242(1):48–55. 10.1016/j.atherosclerosis.2015.06.045 26177274

[48] Königstein K Büschges JC Sarganas G Krug S Neuhauser H Schmidt-Trucksäss A. Exercise and carotid properties in the young-the KiGGS-2 study. Front Cardiovasc Med. (2021) 8:767025. 10.3389/fcvm.2021.767025 35071349 PMC8766972

